# Preparation of MAZ-Type Zeolite with High Silica

**DOI:** 10.3390/molecules29143315

**Published:** 2024-07-14

**Authors:** Songcheng Bo, Kaixuan Yang, Hongying Lü, Zhiguo Zhu

**Affiliations:** College of Chemistry and Chemical Engineering, Yantai University, 30 Qingquan Road, Yantai 264005, China; bsc17865561069@163.com (S.B.); hylv@ytu.edu.cn (H.L.)

**Keywords:** MAZ-type zeolite, acid treatment, steaming treatment, interzeolite transformation, high silica

## Abstract

The Si/Al molar ratio of MAZ aluminosilicate zeolite prepared by the direct hydrothermal method is generally less than five, thus giving rise to poor thermal and hydrothermal stability for this low-silica zeolite. With the purpose of enhancing the Si/Al molar ratio of MAZ zeolite, post-synthesized methods including acetic acid treatment and steaming treatment, as well as interzeolite transformation from FAU zeolite, were employed to prepare MAZ zeolite with high silica. It was found that steaming treatment was more effective in increasing the Si/Al molar ratio in comparison with acetic acid treatment, affording a maximum Si/Al molar ratio of 16.9 along with a preserved crystallinity of approximately 75%. Additionally, high-silica MAZ zeolite with a Si/Al molar ratio of up to 7.3 was also capable of being directly hydrothermally synthesized using interzeolite transformation from FAU zeolite.

## 1. Introduction

Zeolite is a kind of porous nano-inorganic material with high crystallinity, regular pore structure, and high thermal and hydrothermal stability [1,2,3,4,5,6]. FAU, ZSM-5, MOR, and Beta zeolites have been widely used in petroleum refining, petrochemical, and other fields such as electronics, metallurgy, and environmental protection due to their excellent solid acid properties and outstanding shape-selective performance [3,7,8,9]. However, Mazzite zeolite, which was successfully synthesized at a similar age to the above-mentioned zeolite, has not been employed on the industrial scale. Mazzite is also called needle zeolite, and its topology code is termed MAZ. Synthetic Mazzite zeolites include ZSM-4 and omega, which were prepared at the same time by Mobil Oil and Union Carbide, respectively [10,11]. Galli et al. resolved the structure of Mazzite zeolite [12], which was composed of a decahedral sodium rhombic zeolite cage, belonging to the P63/mmc space group, and the cell constant was as follows: a = 1.84 nm, b = 1.84 nm, c = 0.76 nm, α = 90.0°, β = 90.0°, and γ = 120.0° [13,14]. Mazzite zeolite contains a 12-membered ring (0.74 nm) and a twisted 8-membered ring (0.34 × 0.56 nm). Therefore, the diffusion and reaction of most organic molecules can only take place in the large 12-membered channel systems [8,15,16,17,18].

Under the same Si/Al molar ratio, the acidity of MAZ zeolite is significantly stronger than that of ZSM-5, Modenite, and FAU zeolite [19,20,21,22]. Its acidity is considered to be the strongest, which can catalyze reactions requiring Brønsted active centers with strong acidity, such as toluene conversion, hydrocracking, alkylation, isomerization, methane-to-methanol, etc. [19,23,24,25,26,27]. In general, MAZ zeolite is hydrothermally synthesized from silica-alumina gel with the organic-structure-directing agents of tetramethylammonium hydroxide, pyrrolidine, choline, etc. [16,28,29,30]. Normally, the Si/Al molar ratio of MAZ zeolites prepared by the direct hydrothermal method is less than five, and the crystallinity decreases seriously after high-temperature calcination (above 600 °C), resulting in structure collapse (poor thermal stability), which limits its application in petroleum refining or catalytic cracking process [28,30,31,32]. Consequently, it is an urgent problem to improve the Si/Al ratio of MAZ zeolite by novel methods. In this manuscript, the Si/Al ratio of MAZ zeolite was enhanced by a post-treatment method and the direct hydrothermal synthesis strategy of interzeolite transformation on the premise of preserving the crystallinity to a certain extent. In addition, the hydrothermal synthesis process of MAZ zeolite was also investigated in detail.

## 2. Results and Discussion

### 2.1. Hydrothermal Synthesis of MAZ Zeolite

**Figure 1:**
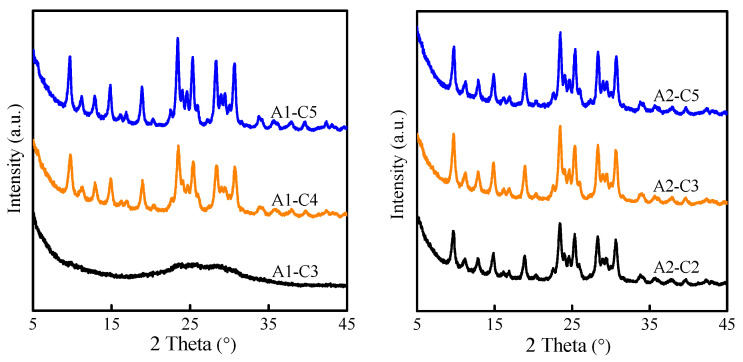
XRD patterns of solid products obtained from different aged and crystallized times.

MAZ zeolite was hydrothermally synthesized in a SiO_2_-Al_2_O_3_-Na_2_O-TMAOH-H_2_O system using silica sol, sodium aluminate, and TMAOH as silicon source, aluminum source, and template agent, respectively. The synthesis process was divided into two steps of aging and crystallization. As can be seen from Figure 1, full crystallization time was closely related to aging time. Except for the A1-C3 sample, all the other samples showed characteristic diffraction peaks at 9.7, 11.2, 12.9, 14.9, 18.9, 23.5, 25.3, 28.3, and 30.7°, assigned to MAZ zeolite (JCPD No. 23-1894) [19]. When the aging time was 1 and 2 days, the time required for complete crystallization of MAZ zeolite was 5 and 3 days, respectively. Considering energy consumption and synthesis period, the optimal aging time and crystallization were fixed to 2 and 3 days, respectively.

The zeolite yields of A1-C5 and A2-C3 samples were about 80 wt% according to the dry mass of SiO_2_ and Al_2_O_3_ (zeolite yield = *M*_product_/*M*_gel_ × 100%).

**Figure 2:**
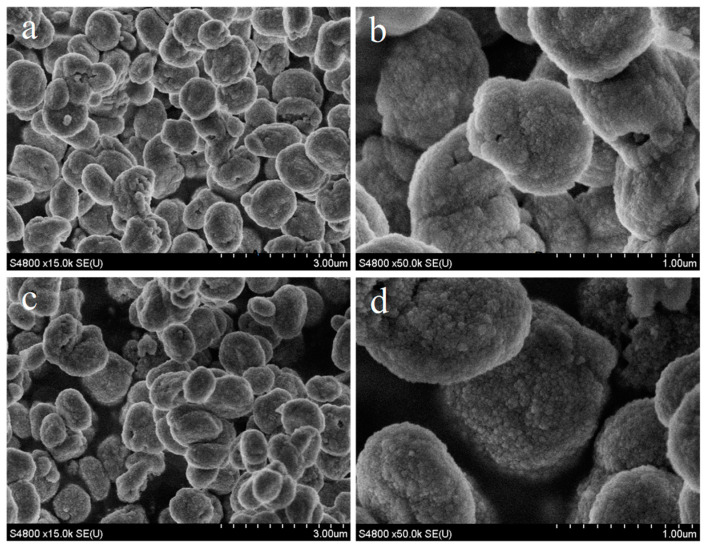
SEM images of A1-C5 (**a**,**b**) and A2-C3 (**c**,**d**) samples.

As exhibited in Figure 2, the A1-C5 and A2-C3 samples all showed ellipsoidal morphology formed by agglomeration of nanoparticles. The mean sizes of the A1-C5 and A2-C3 samples were 0.98 and 1.00 μm, respectively (Figure S1). The particle size distribution of the A1-C5 and A2-C3 samples was 0.67–1.37 and 0.60–1.27 μm, respectively. Therefore, they possessed comparable particle mean size and size distribution. This result indicates that the morphology and particle size of MAZ zeolite are not directly associated with the synthesis period.

**Figure 3:**
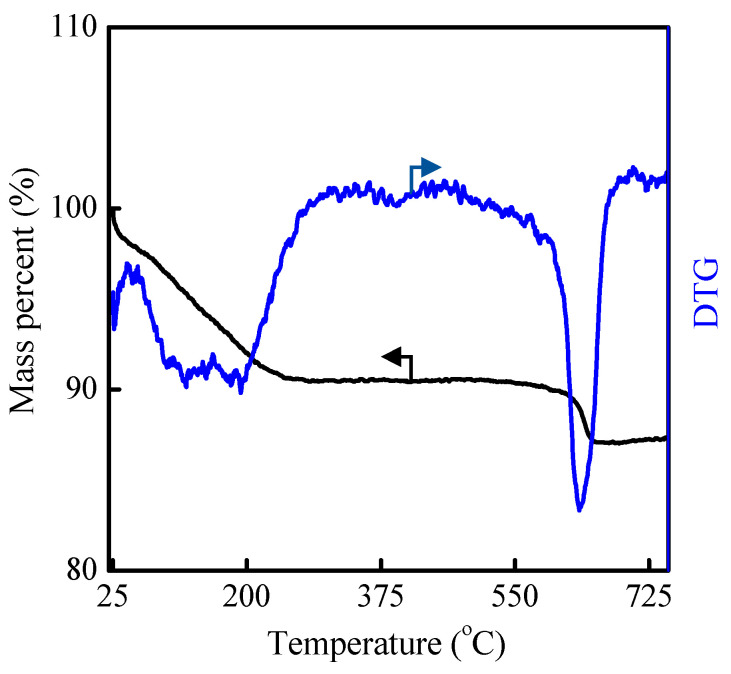
TG and DTG curves of as-prepared A2-C3 sample.

Next, the TG test was performed for the as-synthesized A1-C5 and A2-C3 samples (Figure 3 and Figure S2). The weight loss before 250 °C and between 250 and 750 °C was attributed to the mass of adsorbed water and the content of organics in the raw powder, respectively [8,33]. As for both as-synthesized MAZ samples, the decomposition temperature of organics was in the region of 550–650 °C. The content of adsorbed water and organics was about 10 and 4 wt%, respectively, which was consistent with those in MAZ zeolite prepared by other methods [8,23].

### 2.2. Preparation of MAZ Zeolite with High Silica by Post-Treated Route

After inductively coupled plasma (ICP) analysis, the Si/Al molar ratio of the above-prepared H-MAZ was 4.0. If it was treated directly with a strong acid solution, its framework structure would collapse completely (Figures S3 and S4). In order to improve the Si/Al molar ratio of MAZ zeolite while maintaining the framework structure, the following two methods were adopted.

**Figure 4:**
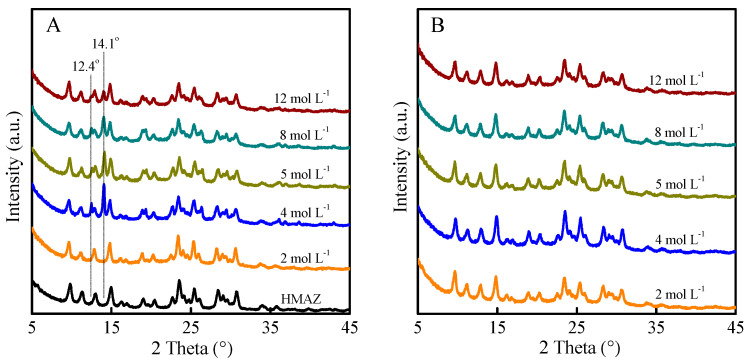
XRD patterns of (**A**) acetic-acid-treated H-MAZ zeolite and (**B**) subsequent hydrochloric acid treatment in ethanol solution.

The first method was to treat the HMAZ with different concentrations of acetic acid followed by hydrochloric acid treatment in ethanol solution. As shown in Figure 4A, when the acetic acid concentration was 2 mol L^−1^, the framework structure of the treated MAZ remained intact compared with the parent HMAZ. With the increase in acetic acid concentration, two sharp diffraction peaks appeared at 12.4 and 14.1°, which may be attributed to aluminum acetate. With the concentration of 4 mol L^−1^ acetic acid, the diffraction peaks at these two positions were the strongest, indicating that the dealumination degree was the most serious. It is worth noting that the zeolite skeleton structure can be well maintained after acetic acid treatment. After these acetic-acid-treated samples were further subjected to hydrochloric acid treatment in ethanol solution, the diffraction peaks of 12.4 and 14.1° completely disappeared, suggesting that hydrochloric acid treatment eliminated the aluminum species in the extra-framework position. Finally, when the concentration of acetic acid was 4 mol L^−1^, the Si/Al molar ratio of the dealuminated MAZ zeolite treated by the combination of acetic acid and hydrochloric acid was 7.5, implying the removal of 47% skeleton aluminum in the parent HMAZ. 

**Figure 5:**
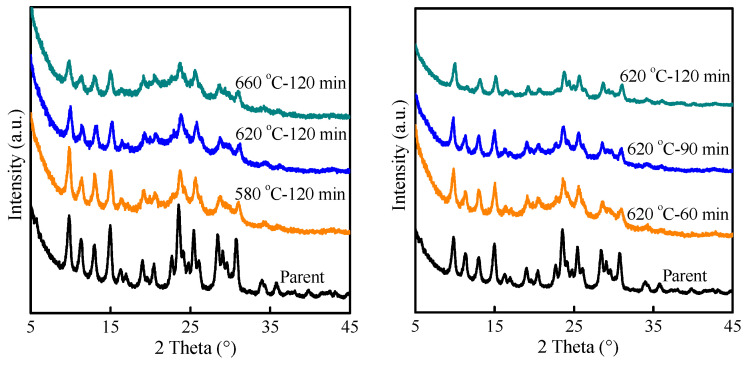
XRD patterns of HMAZ treated by steaming under different conditions.

The second method was that HMAZ zeolite was performed by the integration of steaming and HNO_3_ treatment. First, the parent HMAZ zeolite was treated with 100% water vapor at different temperatures for various times. As shown in Figure 5, the zeolite crystallinity decreases to some extent, which may be due to the existence of structural rearrangement and Si^4+^ migration during steaming treatment [32]. Then, the extra-framework aluminum was removed by treating it with dilute nitric acid at 80 °C for 2 h. The effect of the acid leaching and the steaming treatment was exhibited in Table 1. With the increase in steaming treatment temperature, the higher steaming treatment temperature contributed to an increase in the Si/Al molar ratio in the final sample, but a decrease in zeolite crystallinity. For instance, the Si/Al molar ratio and crystallinity were 11.8 and 77.9%, respectively, with a temperature of 620 °C and a time of 2 h. Considering the Si/Al molar ratio and crystallinity, 620 °C was selected as the optimal steaming treatment temperature. At 620 °C, further shortening the steaming treatment time, the final Si/Al molar ratio decreased, but the crystallinity slightly increased. The final Al content and crystallinity can also be modulated by adjusting the concentration of nitric acid. With nitric acid of 2 mol L^−1^, the Si/Al molar ratio and crystallinity were 16.9 and 74.4%, respectively, implying that 76% Al was dislodged from the HMAZ zeolite.

The morphology was not significantly changed after acid treatment (Figure S5). Nonetheless, the obvious transformation was observed in the morphology for steaming treatment. The large micro-size particles were decomposed into numerous irregular small particles with a size of 200–800 nm. As shown in Table S1, the parent MAZ zeolite possessed a specific surface area of 412 m^2^ g^−1^, microporous volume of 0.13 cm^3^ g^−1^, and mesoporous volume of 0.11 cm^3^ g^−1^. Parent MAZ zeolite showed ellipsoidal morphology formed by agglomeration of nanoparticles (Figure 2). The nanoparticles may provide these interparticle mesopores. After acid treatment and steaming, the microporous volume decreased, perhaps derived from their decrease in crystallinity. In particular, the mesoporous volume increased significantly after steaming likely due to the disassembly of large crystal particles (Figure S5).

### 2.3. Preparation of MAZ Zeolite with High Silica by Interzeolite Transformation

**Figure 6:**
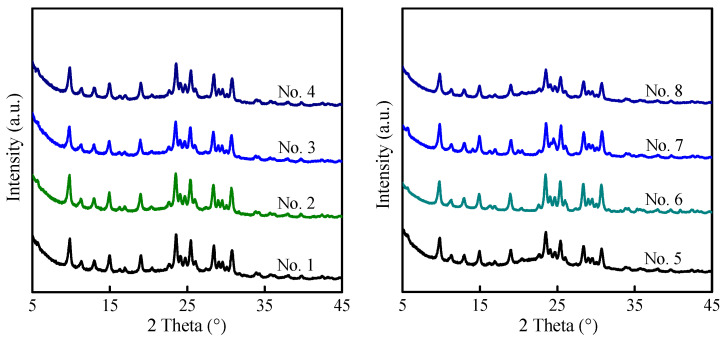
XRD patterns of HMAZ treated by steaming under different conditions.

Interzeolite transformation was capable of promoting zeolite nucleation and crystal growth by altering the aggregation state of the silicon source in the synthesis gels [34,35,36]. Inspired by this, MAZ zeolite was hydrothermally synthesized by the interzeolite transformation of FAU zeolite without the assistance of organic structure-directing agents. As shown in Figure 6, MAZ zeolite was obtained without any impurities. When using USY with the Si/Al molar ratio of 6−44 as a silica source (Nos. 1−7, Table 2), their crystallinity was comparable. When the Si/Al molar ratio reached up to 168 for pristine FAU zeolite, the crystallinity decreased sharply. In addition, with the increase in the Si/Al molar ratio for FAU zeolite, the obtained MAZ zeolite demonstrated higher a Si/Al molar ratio but a rapid decrease in product yields (Table 2). If the seed amounts were enhanced, the Si/Al molar ratio of MAZ zeolite hardly changed; meanwhile, the product yield was slightly improved. When LiOH was replaced by NaOH as an alkali source, both the Si/Al molar ratio and product yield increased significantly. As for LiOH as an alkali source and a Si/Al molar ratio of 168 for FAU zeolite, the Si/Al molar ratio of MAZ zeolite was as high as 7.3, but its product yield was only 8%. Consequently, the Si/Al molar ratio and product yields were two contradictory parameters. Unexpectedly, the Si/Al molar ratio of MAZ zeolite was indeed enhanced up to 7.3 through the interzeolite transformation strategy in comparison with that (generally below five) by the conventional hydrothermal method. Hydrothermal stability was also tested by water steaming treatment at 750 °C for 2 h. As shown in Figure S6, the framework structure of MAZ with a Si/Al ratio of 7.5 and 6.4 nearly collapsed (Figure S6a,c). However, the framework structure of MAZ with a higher Si/Al ratio of 16.9 was maintained to a certain extent (Figure S6b).

## 3. Materials and Methods

### 3.1. Materials

Tetramethylammonium hydroxide (TMAOH, 25 wt%), silica sol (30 wt%), sodium hydroxide (NaOH, 96 wt%), sodium aluminate (99 wt%), ammonium chloride (NH_4_Cl, 99.8 wt%), ethanol (95 wt%), lithium hydroxide (56.5 wt%), and hydrochloric acid (36.0 wt%) were purchased from Sinopharm Group Chemical Reagent Co., Ltd. (Shanghai, China). Ultrastable Y (USY) zeolite (Si/Al = 6 and 19) was supplied by Shanghai Xinnian Petrochemical Auxiliary Co. Ltd. (Shanghai, China).

### 3.2. Hydrothermal Synthesis of MAZ Zeolite

MAZ zeolite with low silica was prepared by the hydrothermal method according to reference with some changes [8]. The synthesis process was as follows: 0.0571 g sodium hydroxide, 0.0820 g sodium aluminate, and 0.0875 g tetramethylammonium hydroxide were added into 0.2244 g deionized water one by one and stirred at room temperature for 30 min. Then, 1.0 g silica sol was further added to the mixed solution. After stirring for 10 min, the final gel molar composition was SiO_2_:0.1 Al_2_O_3_:0.24 Na_2_O:0.048 TMAOH:11.0 H_2_O. The vessel containing synthesis gel was sealed completely. After aging at 25 °C for a certain time (1 or 2 days), the gel was transferred to a Teflon-lined autoclave and statically crystallized at 100 °C for a certain time (2 to 5 days). After crystallization was completed, the autoclave was removed from the oven and cooled down with water for 15 min. Then, the product was filtered and washed with deionized water (300 mL) three times. The sample was dried at 80 °C for 8 h, and then placed in the Muffle furnace and kept at 550 °C for 6 h using a heating rate of 2 °C min^−1^ under air atmosphere. The resultant sample was named as A*x*-C*y*, where *x* and *y* represented aging and crystallization time in the unit of day, respectively. 

H-type samples were prepared from calcined Na-type MAZ by ammonium exchange. The A2-C3 samples were subjected to ion exchange in 1 mol L^−1^ NH_4_Cl solution with a solid-to-liquid mass ratio of 1:20 at 80 °C for 2 h, and then filtrated with deionized water repeatedly and dried at 80 °C for 8 h. The process was repeated two more times to obtain NH_4_-type MAZ. H-MAZ was obtained by calcinating it at 550 °C for 6 h using a heating rate of 2 °C min^−1^ under air atmosphere.

### 3.3. Post-Treated Dealumination of H-MAZ Zeolite

#### 3.3.1. Acetic Acid Treatment followed by Hydrochloric Acid Treatment in Ethanol Solution

H-MAZ was placed in acetic acid solution with different concentrations (1 g zeolite:20 g acetic acid solution), treated at 120 °C for 10 h, and then washed with deionized water repeatedly and dried at 80 °C for 8 h. Then, the acetic-acid-treated sample was further performed with 1 mol L^−1^ hydrochloric acid in ethanol solution (1 g zeolite:20 g solution) at room temperature for 2 h, which was then filtrated with deionized water repeatedly and dried at 80 °C for 8 h.

#### 3.3.2. Steaming Treatment

H-MAZ was treated with 100% water vapor at various temperatures (580−620 °C) for different times (60−120 min), and further treated with diluted nitric acid (0.5−2 mol L^−1^) at 80 °C for 2 h to remove zeolite extra-framework aluminum. The obtained mixture was washed with deionized water repeatedly and dried at 80 °C for 8 h.

### 3.4. Interzeolite Transformation of FAU to MAZ Zeolite

MAZ zeolite was prepared by interzeolite transformation of USY zeolite with different molar ratios of Si to Al. The dealuminated process of USY zeolite (Si/Al = 6) is as follows: First, H-USY zeolite was calcined at 600 °C for 6 h. Then, zeolite powder was heated at 130 °C in 6 mol L^−1^ nitric acid aqueous solution with a solid-to-liquid ratio of 1 g zeolite:20 mL nitric acid solution. After treatment at different times (1 and 20 h), this mixture was washed with deionized water for several times, and dried at 100 °C for 10 h. The obtained white solid powder was labeled as USY-*n*, where *n* represents the Si/Al molar ratio of dealuminated USY zeolite. USY-44 and USY-168 zeolite were obtained by acid treatment for 1 and 20 h, respectively.

MAZ zeolite was hydrothermally synthesized using USY-*n* as a silicon source and aluminum source. Briefly, 0.3 g USY-*n*, 0.075 g, or 0.1 g as-synthesized MAZ seed (A2-C3 sample) was added into the alkali solution pre-prepared by dissolving 0.1250 g NaOH or 0.1274 g LiOH into 1.35 g deionized water. After stirring for 30 min, the gel was then transferred to a Teflon-lined autoclave and crystallized at 70 °C for 17 days under static condition. Then, the autoclave was taken from the oven, and cooled down with water for 15 min. The product was washed with deionized water repeatedly, dried at 80 °C for 8 h, and calcined at 550 °C for 6 h with a heating rate of 2 °C min^−1^ under air atmosphere.

### 3.5. Characterizations

The phase and crystallinity of the molecular sieve were characterized by X-ray powder diffractometer (XRD) on Rigaku Smart Lab3 diffractometer with a Cu-Kα radiation (λ = 1.5405 Å, 35 kV, 25 mA). Scanning electron microscopy (SEM) was performed on Hitachi S-4800 microscopy to detect zeolite morphology and crystal size. The thermogravimetric analyses (TG) were carried out on a METTLER TOLEDO TGA/SDTA851e apparatus from room temperature to 750 °C with a heating rate of 10 °C min^−1^ in air. The contents of Si and Al in zeolites were analyzed by ICP on a Thermo IRIS Intrepid II XSP atomic emission spectrometer. N_2_ adsorption–desorption isotherms were recorded at −196 °C on a Micromeritics ASAP2020 PLUS HD 88 instrument after activating the samples under vacuum at 300 °C for 6 h.

## 4. Conclusions

MAZ zeolite with high silica content was prepared by both post-treatment and direct hydrothermal synthesis. Acetic acid and steaming treatment can improve the Si/Al molar ratio of MAZ zeolite. But the former was relatively limited, steaming treatment can dramatically improve the Si/Al molar ratio in MAZ zeolite. When the crystallinity was approximately 75%, the Si/Al molar ratio can reach 16.9. In addition, MAZ zeolite with high silica can also be directly synthesized by interzeolite transformation from FAU zeolite. The product yield and Si/Al molar ratio were closely associated with the initial Si/Al molar ratio of FAU zeolite, the type of alkali source, and the amounts of seeds, and the highest Si/Al molar ratio can achieve 7.3.

## Figures and Tables

**Table 1:** Effect of the acid leaching performed after the steaming treatment.

No.	Steaming Treatment		HNO_3_ Acid Leaching			Bulk Si/Al ^a^	Crystallinity (%)
	T (°C)	Time (h)	Concentration (M)	Time (h)	T (°C)		
1	580	2	1	2	80	10.4	85.7
2	620	2	1	2	80	11.8	77.9
3	660	2	1	2	80	15.7	64.5
4	620	1	1	2	80	8.3	86.6
5	620	1.5	1	2	80	9.7	83.0
6	620	2	0.5	2	80	7.2	81.1
7	620	2	2	2	80	16.9	74.4

^a^ Analyzed by ICP technique.

**Table 2:** Seed-assisted and template-free synthesis of high-silica MAZ from FAU zeolite ^a^.

No.	Synthesis Conditions			Product		
	Si/Al Ratio of Starting FAU	OH Source	Seed (wt%)	Phase	Yield (%)	Bulk Si/Al ^b^
1	6	LiOH	0.33	MAZ	78	4.8
2	19	LiOH	0.25	MAZ	39	6.4
3	19	LiOH	0.33	MAZ	34	6.7
4	19	NaOH	0.33	MAZ	22	4.2
5	44	LiOH	0.33	MAZ	15	6.3
6	44	NaOH	0.25	MAZ	11	3.4
7	44	NaOH	0.33	MAZ	12	3.6
8	168	LiOH	0.33	MAZ	8	7.3

^a^ Synthesis time = 17 days, Si/Al ratio of non-calcined MAZ seeds = 4.0, H_2_O/SiO_2_ = 15, OH^−^/SiO_2_ = 0.6, 70 °C, static. ^b^ Analyzed by ICP technique.

## Data Availability

The data presented in this study are available on request from the author.

## Supplementary Materials

The following supporting information can be downloaded at: https://www.mdpi.com/article/10.3390/molecules29143315/s1, Figure S1: Particle size distribution of MAZ samples. Figure S2: TG and DTG curves of as-prepared A1-C5 sample. Figure S3: XRD pattern of HNO_3_ treated MAZ zeolite. Figure S4: XRD pattern of hydrochloric acid treated MAZ zeolite. Figure S5: SEM images of MAZ zeolite with (a) Si/Al molar ratio of 7.5 obtained by acid treatment and (b) with Si/Al molar ratio of 16.9 obtained by steaming treatment. Figure S6: XRD patterns of MAZ zeolite after steaming at 750 °C for 2 h with (a) initial Si/Al molar ratio of 7.5 obtained by the combination treatment of acetic acid and hydrochloric acid, (b) with initial Si/Al molar ratio of 16.9 obtained by steaming treatment, and (c) with initial Si/Al molar ratio of 6.4 obtained by interzeolite transformation. Table S1: Textural properties of MAZ zeolites after acid treatment and steaming.
