# Supplementary material for: Preparation of MAZ-Type Zeolite with High Silica
Source: Molecules. 2024 Jul 14;29(14):3315. doi: 10.3390/molecules29143315 (PMC11279715; doi:10.3390/molecules29143315)
Supplement: Supplementary file 1 [file molecules-29-03315-s001.zip › molecules-3085123-supplementary.pdf]

Preparation of MAZ-Type Zeolite with High Silica

Songcheng Bo, Kaixuan Yang *, Hongying Lü and Zhiguo Zhu *

College of Chemistry and Chemical Engineering, Yantai University, 30 Qingquan Road, Yantai 264005, China; bsc17865561069@163.com (S.B.); hylv@ytu.edu.cn (H.L.)
* Correspondence: yangkaixuanyt@ytu.edu.cn (K.Y.); zhuzg@ytu.edu.cn (Z.Z.)

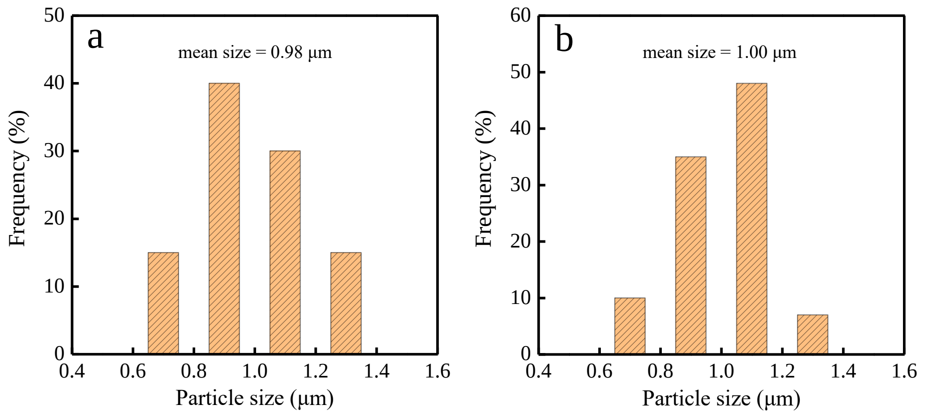

Figure S1. Particle size distribution of (a) A1-C5 and (b) A2-C3 MAZ samples.

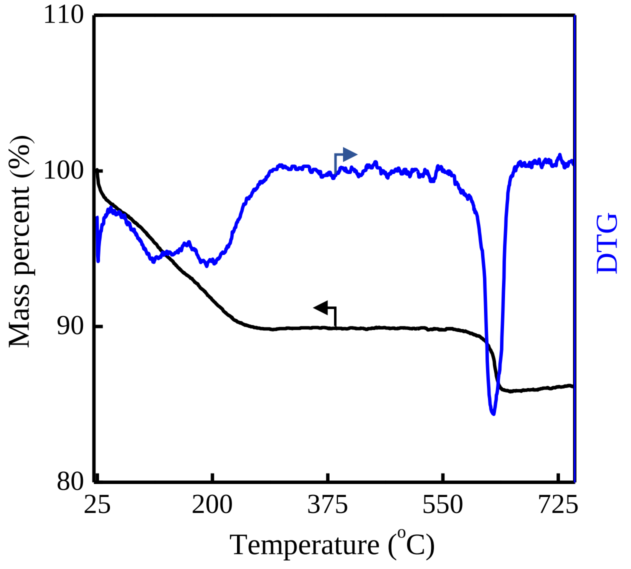

Figure S2. TG and DTG curves of as-prepared A1-C5 sample.

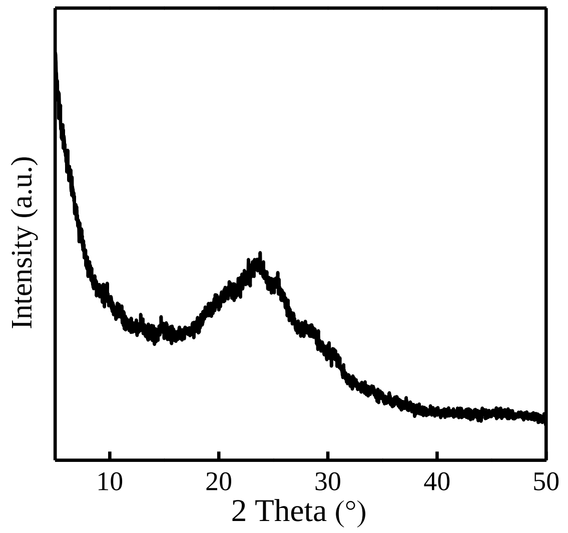

Figure S3. XRD pattern of HNO_3 treated MAZ zeolite. Treatment conditions: HNO_3, 0.2 mol L^{-1}; Solid-to-liquid, 1 g: 20 mL; temperature, 80 ^{o}C, time, 2 h.

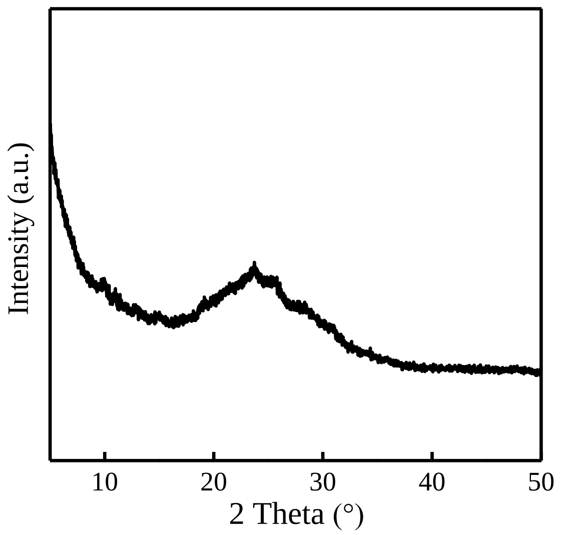

Figure S4. XRD pattern of hydrochloric acid treated MAZ zeolite. Treatment conditions: hydrochloric acid, 0.2 mol L^{-1}; Solid-to-liquid, 1 g: 20 mL; room temperature, time, 2 h.

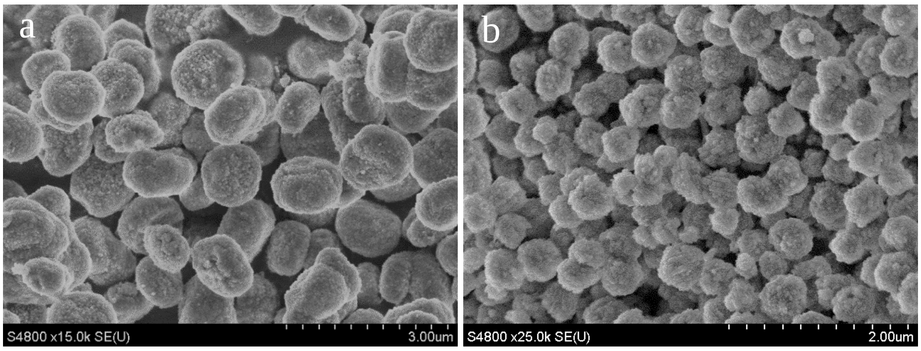

Figure S5. SEM images of MAZ zeolite with (a) Si/Al molar ratio of 7.5 obtained by acid treatment and (b) with Si/Al molar ratio of 16.9 obtained by steaming treatment.

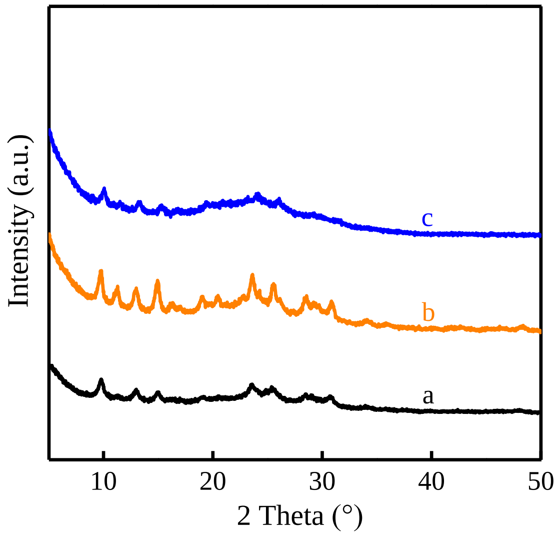

Figure S6. XRD patterns of MAZ zeolite after steaming at 750 °C for 2 h with (a) initial Si/Al molar ratio of 7.5 obtained by the combination treatment of acetic acid and hydrochloric acid, (b) with initial Si/Al molar ratio of 16.9 obtained by steaming treatment, and (c) with initial Si/Al molar ratio of 6.4 obtained by interzeolite transformation.

Table S1 Textural properties of MAZ zeolites after acid treatment and steaming.

No.	Sample	Si/Al[a]	S_{BET}[b] (m^2 g^{-1})	Pore volume (cm^3 g^{-1})		
				V_{total}[c]	V_{micro}[d]	V_{meso}[e]
1	MAZ	4.0	412	0.24	0.13	0.11
2	MAZ-acid treatment	7.5	393	0.23	0.11	0.12
3	MAZ-steaming	16.9	489	0.28	0.09	0.19

[a] Analyzed by ICP technique.

[b] Specific surface area (S_{BET}), estimated by N_2 adsorption at -196 ℃ using the BET method.

[c] Total pore volume (V_{total}), as calculated from the adsorption capacity at $P/P_0 = 0.95$.

[d] Microporous volume (V_{micro}), as determined by the *t*-plot method.

[e] Mesoporous volume (V_{meso}), $V_{meso} = V_{total} - V_{micro}$.
